# Sex-determining region Y (SRY) attributes to gender differences in *RANKL* expression and incidence of osteoporosis

**DOI:** 10.1038/s12276-019-0294-3

**Published:** 2019-08-14

**Authors:** Klemen Kodrič, Janja Zupan, Tilen Kranjc, Radko Komadina, Vid Mlakar, Janja Marc, Nika Lovšin

**Affiliations:** 1 University of Ljubljana, Faculty of Pharmacy, The Chair of Clinical Biochemistry, Aškerčeva cesta 7, 1000 Ljubljana, Slovenia; 2 General and Teaching Hospital Celje, Department for Research and Education, Celje, Slovenia

**Keywords:** Osteoporosis, Genetics, Metabolic bone disease

## Abstract

Receptor activator of nuclear factor *κ*B ligand (RANKL) plays a crucial role in bone metabolism. *RANKL* gene misregulation has been implicated in several bone and cancer diseases. Here, we aimed to identify novel transcription regulators of *RANKL* expression. We discovered that transcription factors, sex-determining region Y (SRY) and c-Myb, regulate *RANKL* expression. We demonstrated that c-Myb increases and male-specific SRY decreases *RANKL* expression through direct binding to its 5’-proximal promoter. These results are corroborated by the gene expression in human bone samples. In osteoporotic men, expression of *RANKL* is 17-fold higher, which correlates with the drastically reduced expression (200-fold) of *Sry*, suggesting that in osteoporotic men, the upregulation of *RANKL* is caused by a decrease of *Sry*. In healthy men, the expression of *RANKL* is 20% higher than that in healthy women. Our data suggest that gender differences in *RANKL* expression and bone quality could be due to the sex-specific transcription factor SRY.

## Bone health: Insight into gender differences in osteoporosis

A male-specific gene offers clues to diagnosis and treatment of age-related osteoporosis. Osteoporosis was known to be linked to higher expression levels of *RANKL*, a gene that induces bone resorption, but the details were poorly understood. Nika Lovsin at the University of Ljubljana in Slovenia and co-workers searched for the genetic switches that control *RANKL* levels. They found that *SRY*, a gene on the male-specific Y chromosome, was a strong repressor of *RANKL*. In bone samples from osteoporotic men, expression levels of *SRY* levels were low and those of *RANKL* were high, suggesting that in men, when *SRY* fails to keep the bone-resorbing *RANKL* in check, osteoporosis results. *SRY* shows promise as an osteoporosis marker in men, or for development of treatment for both genders. Future research could address what triggers decreased SRY expression in men.

## Introduction

Receptor activator of nuclear factor κB ligand (RANKL) is part of the RANKL/RANK/OPG system and is one of the most important molecules in bone remodeling. RANKL was identified as a key signaling molecule for the differentiation of bone macrophages into mature osteoclasts and for the activation of these cells, which leads to bone resorption. RANKL functions by binding to the RANK receptor on osteoclasts. RANKL belongs to the tumor necrosis factor (TNF) family of cytokines and was also named TNFSF11^1^. RANKL is found as membrane-bound and soluble forms, the latter of which is produced by alternative splicing or by proteolytic cleavage of membrane-bound RANKL^2–4^. Osteoprotegerin (OPG) is a RANKL decoy receptor that can bind RANKL and prevent its function^5,6^. In addition to cells involved in bone formation (e.g., osteoblasts, their precursors, and osteocytes), there are other cells that express RANKL, such as T and B lymphocytes, mammary epithelial cells, vascular endothelial cells, synovial fibroblasts, chondrocytes, keratinocytes, malignant cells, cells in periodontal tissue, and other cell types^7^. Studies have shown that RANKL is involved in numerous physiological processes, as well as bone metabolism, such as in T cell functions and lymph node and mammary gland development^5,8–10^. In addition, RANKL is involved in pathological processes, such as the migration of tumor cells and the development of bone metastases in diseases, such as breast, lung and prostate cancer^11–15^.

While the biological functions of RANKL have been extensively studied, much less is known about the regulation of the *RANKL* gene at the transcription level. RANKL is regulated by transcription factors that bind to distal and proximal regulatory regions. The major expression regulator of RANKL is parathyroid hormone (PTH), which acts on the 76,000-bp-distant cAMP-response elements (CREs) through the protein kinase A–cAMP pathway, which requires CREB^7,16–21^. CREB is also required for 1,25(OH)_2_D_3_ and oncostatin M (OSM)-stimulated *RANKL* expression^19^. In addition to the binding of CREB, the same DNA region also contains a RUNX2 binding site. These binding sites and their importance were confirmed with electrophoretic mobility shift assays (EMSAs) and ChIP and transgenic mouse models^16,19,22^. The same distant enhancer also contains 1,25(OH)_2_D_3_ response elements–the VDREs^19,22,23^. VDREs were also found at −96, −87, −75, −25, and −20 kb upstream of the transcription start site^23–25^. Other known *RANKL* transcription regulators acting through distal regulatory regions are interleukin (IL)-6-type cytokines^26,27^, molecules of the Wnt/β-catenin signaling pathway^28–35^ and c-Fos in activated T cells^36^. Previous studies have indicated that Vitamin D, PTH, and other transcriptional regulators could also regulate *RANKL* gene expression through its proximal promoter^37,38^. A recent study revealed the involvement of C/EBPβ in the transcriptional regulation of *RANKL* through binding to a CCAAT-box in the region −56/−51 upstream of the transcription start site of the *RANKL* gene^39^.

Overall, very little is known about the factors that regulate *RANKL* expression at the 5’-proximal region of the gene. The aim of the present study was therefore to identify other transcription factors that bind to this 5’-proximal region and regulate the expression of *RANKL* and to evaluate their in vivo significance. Two novel transcription factors were identified here: SRY and c-Myb. SRY is encoded by the sex determining region Y (*Sry*) gene that initiates testis development. SRY is implicated in sex determination and belongs to the SRY-box (*Sox*) gene family. SRY is not expressed in females, and this protein is responsible for the development of the male phenotype in therian mammals^40^. c-Myb is a transcription factor that has previously been associated with bone formation and chondrogenesis^41–43^. We hypothesized that SRY-mediated RANKL gene expression might be the molecular mechanism underlying the sex differences in bone metabolism and bone diseases, such as osteoporosis.

## Materials and methods

### Patients

Bone tissue samples from 112 Slovenian patients were included in this study^44^. Fifty-eight patients (46 female, 12 male) were undergoing hip arthroplasty because of low energy femoral neck fracture (osteoporosis; OP), and 42 patients (29 female, 13 male) had primary hip osteoarthritis (osteoarthritis; OA). Twelve bone tissue samples were collected from male autopsy subjects (control; CTL). All subjects included in the study signed written informed consent prior to inclusion. The study was approved by the Republic of Slovenia National Medical Ethics Committee (consent numbers: KME 91-93/08/10, KME 106/03/06, KME 137/03/06, KME 45/10/16, KME 91/08/10, KME 45/10/16**)** and is summarized in Supplementary Table 1. Detailed descriptions of the patient samples have been reported previously^44^. Briefly, the exclusion criteria for all of these patients included a history of systemic or metabolic diseases that impact bone or mineral metabolism or taking any drugs known to impact bone or mineral metabolism in the 12 months prior to the surgery. Patients with any diseases or drug use that might influence bone metabolism were excluded from the study.

### Cell experiments

Human bone osteosarcoma (HOS) cells (ATCC CRL-1543) were grown in Dulbecco’s modified Eagle’s medium (DMEM) (Gibco, Thermo Fisher Scientific, Massachusetts, USA) supplemented with 10% fetal bovine serum (FBS) (Gibco, Thermo Fisher Scientific, Massachusetts, USA), 1% L-glutamine (Gibco, Thermo Fisher Scientific, Massachusetts, USA) and 1% antibiotic/ antimycotic (Gibco, Thermo Fisher Scientific, Massachusetts, USA) at 37 °C under 5% CO_2_. Primary human osteoblasts (Promocell, Heidelberg, Germany) were grown in Osteoblast Growth Medium (Ready-to-use; Promocell, Heidelberg, Germany) at 37 °C under 5% CO_2_ and were subcultured according to the manufacturer’s instructions. Briefly, after thawing, the cells were grown until they reached 70 to 90% confluency, and then the cells were subcultured using DetachKit (Promocell, Heidelberg, Germany). The osteoblast growth medium was changed every 2 days to 3 days. All experiments were performed between the third and the fifth passages. The presence of osteoblast markers was confirmed by quantitative real-time polymerase chain reaction (q-PCR) (Supplementary Fig. 1).

### Plasmid construction and site-specific mutagenesis

Ensembl ENST00000398795 (Human GRCh38.p2) was used as the reference sequence for the *RANKL* gene. Five different fragments of the human *RANKL* proximal promoter region were PCR amplified from the genomic DNA with a 5’-primer harboring a BglII site and a reverse primer complementary to the sequences near the transcription start site of the *RANKL* gene harboring a HindIII site. The primers used for PCR amplifications of the fragments are listed in Supplementary Table 1. The resulting PCR fragments were digested with BglII and HindIII and cloned into the pGL3 basic vector (Promega, Madison, USA). With PCR amplification, five fragments of the *RANKL* proximal promoter were obtained, with sizes of 898 bp (pGL3-F4), 762 bp (pGl3-F3), 538 bp (pGl3-F2), 346 bp (pGl3-F1), and 1769 bp (pGl3-F5). Mutants in the putative Sex-determining region Y (Sry) binding site (−732/−726 bp upstream of the *RANKL* transcription start, −731 AA > CC, pGl3-F4-SRY) and putative Myb proto-oncogene C (c-Myb) binding site (691/−674 bp upstream of the *RANKL* transcription start, −680 TT > GG, pGl3-F4-c-Myb) were prepared using a Quick-Change II Site-Directed Mutagenesis kit (Stratagene, San Diego, California, USA) and the primers listed in Supplementary Table 2.

The pCIneo-hc-Myb-HA expression vector was kindly donated by Prof. Odd Stokke Gabrielsen (Department of Biosciences, University of Oslo, Oslo, Norway). The pcDNA3-hSRY expression vector was constructed from the pcDNA3-FLAG-hSRY vector, which was kindly donated by Dr. Vincent Harley (Hudson Institute of Medical Research, Melbourne, Victoria, Australia), through the removal of the FLAG tag by PCR using the primers listed in Supplementary Table 2. The pEGFP-SRY plasmid was constructed by amplifying the *Sry* open reading frame from plasmid pcDNA3-FLAG-hSRY, kindly donated by Dr. Vincent Harley (Prince Henry’s Institute, Australia), using the primers listed in Supplementary Table 1 to add the restriction sites for ligation into the pEGFP-N1 plasmid (Clontech, CA, USA). All constructs were verified by DNA sequencing (GATC, Konstanz, Germany).

### Western blotting

Human bone osteosarcoma cells were seeded onto 10-cm^2^ dishes and transfected with empty pcDNA3 expression vector, Sry-FLAG expression vector, or c-Myb-HA expression vector. The cells were lysed after 24 h and resolved by SDS-PAGE. The proteins were transferred to nitrocellulose membranes, subsequently blocked in 5% milk/TTBS, and incubated overnight with the primary antibodies against FLAG (clone M2; Sigma-Aldrich, St. Louis, Missouri, USA) or HA (H3663; Sigma-Aldrich, St. Louis, Missouri, USA). After incubation with the primary antibodies, the membranes were washed with TTBS and incubated with secondary antibodies bound to HRP for 1 h. Substrate was subsequently added, and the proteins were visualized using UVITEC Alliance. Antibodies against beta-actin (A2228, Sigma-Aldrich, St. Louis, Missouri, USA) were used for loading controls. Whole WB membranes are shown in Supplementary Fig. 2.

### Overexpression of Sry-FLAG and c-Myb-HA in human primary osteoblasts

Male and female human primary osteoblasts were plated in 24-well tissue-culture plates (5 × 10^4^ cells per well), incubated overnight, and transfected with 500 ng pcDNA3, Sry-FLAG, or c-Myb-HA using Lipofectamine 2000 (Thermo Fisher Scientific, Massachusetts, USA), according to the manufacturer’s instructions. RANKL expression was analyzed by q-PCR after 48 h, as described below. At least three biological replicates were analyzed for each gender.

### Electroporation of human primary osteoblasts

Human primary osteoblasts were trypsinized with 0.05% Trypsin/EDTA, resuspended in fresh osteoblast growth medium (Promocell) and counted. Then, 1 × 10^6^ cells per nucleofection were transferred to fresh tubes and centrifuged at 100 × *g* for 5 min. After removing the supernatant, the cell pellets were resuspended in 100 μL Mouse Primary Neurons Nucleofector Solution (Amaxa Biosystems) per transfection, with 2 μg pEGFP-SRY added to the cell suspensions, which were transferred to Amaxa electrode cuvettes and electroporated in Amaxa Nucleofector Device II using program T-030. Immediately thereafter, the cells were diluted in 500 μL Osteoblast Growth Medium supplemented with 20% FBS and seeded onto glass coverslips in 12-well plates. The medium was replaced 2 h after nucleofection with 750 μL osteoblast growth medium supplemented with 10% FBS.

### RNA interference knock-down of gene expression

siRNAs for silencing of Sry (sc-38443), c-Myb (sc-29855) and a negative control, nc (sc-37007), were from Santa Cruz Biotechnology. Here, 30-pmol siRNA (final concentration, 300 nM) was nucleofected into 1 × 10^6^ human primary osteoblasts, as described above. After 24 h and 48 h of nucleofection, the growth medium was changed. Then, at 72 h after nucleofection, the RNA was isolated using PeqGold RNA extraction kits (VWR), reverse transcribed, and subjected to quantitative PCR for the genes of interest. Three biological replicates were analyzed.

### Luciferase reporter assay

Luciferase activity was measured with the dual-luciferase reporter assay system (Promega, Madison, WI, USA) according to the manufacturer’s protocol. Briefly, 3.5 × 10^4^ cells were plated per well in 24-well tissue culture plates. After 24 h, cells were transfected with X-tremeGENE HP DNA Transfection Reagent (Roche Diagnostics, Mannheim, Germany), with 500 ng DNA per well. In the case of human primary osteoblasts, 2 µg of pDNA was nucleofected using the Amaxa System as described above for silencing experiments. A plasmid containing the Renilla luciferase gene was used as an internal control. Luciferase activity was measured at 24 h after transfection (BIO-TEK Synergy HT, Fisher Scientific, Pittsburgh, PA, USA). All experiments were carried out at least three times independently.

### Electrophoretic mobility shift assays

Electrophoretic mobility shift assays were performed using LightShift Chemiluminescent EMSA kits (Thermo Fisher Scientific, Waltham, Massachusetts, USA) according to the manufacturer’s instructions. HOS and HeLa nuclear extracts were prepared using NE-PER Nuclear and Cytoplasmic Extraction Reagents (Thermo Fisher Scientific, Waltham, Massachusetts, USA) according to the manufacturer’s instructions. Double-stranded biotinylated oligonucleotides were prepared by hybridization in a C1000 thermal cycler by stepwise 0.5 °C decreases in the temperature, ranging from 95 °C to 35 °C, over 120 cycles, each 30 s long. The binding reactions between oligonucleotides and SRY/c-Myb were performed in 20 µL reactions containing 2 µL 10× binding buffer, 1 µL 100 mM MgCl_2_, 2 µL Poly(dI-dC), 2 µL 50% glycerol, 3 µL nuclear extract, and 20 fmol hybridized oligonucleotides (c-Myb) or 4 µmol hybridized oligonucleotides (SRY). Control reactions were performed without nuclear extracts. The specificity of the DNA-protein complexes was verified by dilution of the nuclear extracts and by competitive binding with nonbiotinylated DNA or mutated nonbiotinylated DNA. A primary rabbit polyclonal antibody against c-Myb (117635, Abcam, Cambridge, UK) was used to confirm the identity of the protein. For SRY, the mouse monoclonal anti-FLAG clone M2 was used (Sigma-Aldrich, St. Louis, Missouri, USA). The biotinylated oligonucleotides listed in Supplementary Table 1 were used in the EMSA experiments.

After the binding reactions, the samples were loaded onto electrophoretic gels and run for 1 h (100 V), then transferred to nylon membranes (30 min, 100 V), and cross-linked on a transilluminator. Biotin-labeled nuclear probes were detected with Chemiluminescent Nucleic Acid Detection Module kits (Pierce, Thermo Scientific, Wilmington, DE, USA) according to the manufacturer’s instructions.

### Immunofluorescence

Cells were fixed with 3% paraformaldehyde (Sigma Aldrich, MO, USA) in phosphate-buffered saline (PBS) (Sigma Aldrich, MO, USA) at 37 °C with 0.2 μg/mL Hoechst 33342 (Sigma Aldrich, MO, USA) for 20 min. The cells were permeabilized with 0.1% Triton X-100 (Sigma Aldrich, MO, USA) in PBS for 5 min, stained with the primary antibodies anti-cMyb (ab117635, Abcam, Cambridge, UK) for 30 min, washed 3 times with PBS, stained with the secondary antibodies anti-rabbit CF568 (20102, Biotium, Fremont, CA, USA) for 30 min, washed 3 times with PBS, and mounted on glass slides with prolong anti-fade with DAPI (Thermo Fischer Scientific, MA, USA). Images were taken with a laser-scanning confocal microscope (710 META; Carl Zeiss Ltd, Germany) at 40× magnification using ZEN software (Carl Zeiss Ltd).

### Human bone tissue immunohistochemistry and immunofluorescence

Bone samples were collected as described previously^44^. Briefly, trabecular bone from the intertrochanteric region of patients undergoing hemi- or total hip arthroplasty due to low-energy osteoporotic fracture or primary osteoarthritis or post-mortem donors with no musculoskeletal disorders were fixed in neutral buffered formalin for up to 24 h and decalcified with 4% EDTA for 2 to 6 weeks. Wax sections were prepared following a routine procedure at the Institute of Pathology, University of Ljubljana (Slovenia). Sections were dewaxed and rehydrated, and antigen retrieval was performed using 0.01 M sodium citrate buffer (pH 6) for 45 min in a microwave. Immunohistochemistry was performed using mouse-specific and rabbit-specific HRP/DAB detection IHC kits (ab64264; Abcam, Cambridge, UK), according to the manufacturer’s procedure, and TBS with Triton X-100 (Sigma-Aldrich, Steinheim, Germany) for washing. The samples were incubated overnight at 4 °C in a humidified chamber with primary rabbit polyclonal antibodies against c-Myb and SRY (ab117635 and ab135239, respectively; Abcam, Cambridge, UK) diluted 1:100 in TBS buffer. Control slides were prepared for each sample using TBS buffer only. The tissue sections were counterstained with hematoxylin solution (Thermo Shandon, Pittsburgh, USA) and examined under a microscope (BX50; Olympus, Hamburg, Germany). The intensity of the staining in osteoblasts, lining cells and osteocytes was compared between all of the samples by two independent blinded evaluators. Images were acquired using a digital camera (Olympus XC50) and the CellSens Dimension program 1.6.0 (Olympus, Hamburg, Germany).

Immunofluorescence for colocalization studies was performed using the following primary antibodies: SRY ab135239 (1:100) and c-Myb ab226470 (1:500) (both Abcam, Cambridge, UK); CD3 D7A6E (1:50) (Cell Signaling Technology, Danvers, Massachusetts, USA); and CD19 sc-19650 (1:50) and CD3 sc-20047 (1:50) (both Santa Cruz Biotechnology, Dallas, Texas, USA). Primary antibodies were applied overnight. The secondary antibodies of goat anti-rabbit Alexa Fluor 633 and goat anti-mouse Alexa Fluor 488 (1:500) (both Thermo Fisher Scientific Waltham, Massachusetts, USA) were applied for 1 h. The slides were mounted with ProLong Gold Antifade Mountant with DAPI (Thermo Fisher Scientific Waltham, Massachusetts, USA), and images were taken using a laser-scanning confocal microscope (710 META; Carl Zeiss Ltd, Germany) at 40× magnification using ZEN software (Carl Zeiss Ltd).

### Quantitative PCR

The expression of *RANKL* was analyzed using qPCR with RNA samples obtained in gain-of-function and loss-of-function experiments. The RNA was extracted from cells, and the complementary DNAs (cDNAs) were synthesized, with gene expression analyses performed as described below. The expression of *RANKL*, Sry, and c-Myb was also analyzed using qPCR assays with the bone samples of Slovenian patients. RNA was extracted from the bone samples and the complementary DNA (cDNA) synthesized according to our previously described procedure^44,45^. Pairs of oligonucleotides for RANKL, RPLP0, GAPDH, and Sry were designed using the Primer Designing Tool (NCBI) (Supplementary Table 1), while for c-Myb, previously optimized oligonucleotides were used^46^. For qPCR, 5× Hot FirePol EvaGreen qPCR Mix Plus (Solis, BioDyne, Tartu, Estonia) was used, following the manufacturer recommendations, on a LightCycler 480 (Roche Diagnostics, Rotkreuz, Switzerland). All samples were quantified in triplicate. Dilution series of cDNA were prepared to create a relative standard curve, and absolute quantification of the data was performed using the second derivative maximum method (LightCycler 480, software version 1.5; Roche Diagnostics, Rotkreuz, Switzerland). All data were normalized to the internal housekeeping genes of ribosomal protein, large, P0 (RPLP0) or glyceraldehyde 3-phosphate dehydrogenase (GAPDH).

### Statistical analysis and bioinformatics

The TF Search program was used to predict transcription factor binding to specific DNA regions. Matrices for vertebrates and threshold scores of more than 85 were used. The results for the luciferase activities were first transformed by calculating their *z*-scores and then normalized from 0 to 1 using unity scaling. The normalized results were then compared using ANOVA. Gene expression results were logarithmically transformed where needed to obtain normal distributions. The results were compared using Student’s *t*-tests or ANOVA.

## Results

### The region −662/−798 bp in the *RANKL* proximal promoter appears to contain binding sites for the transcription factors

**Fig. 1:**
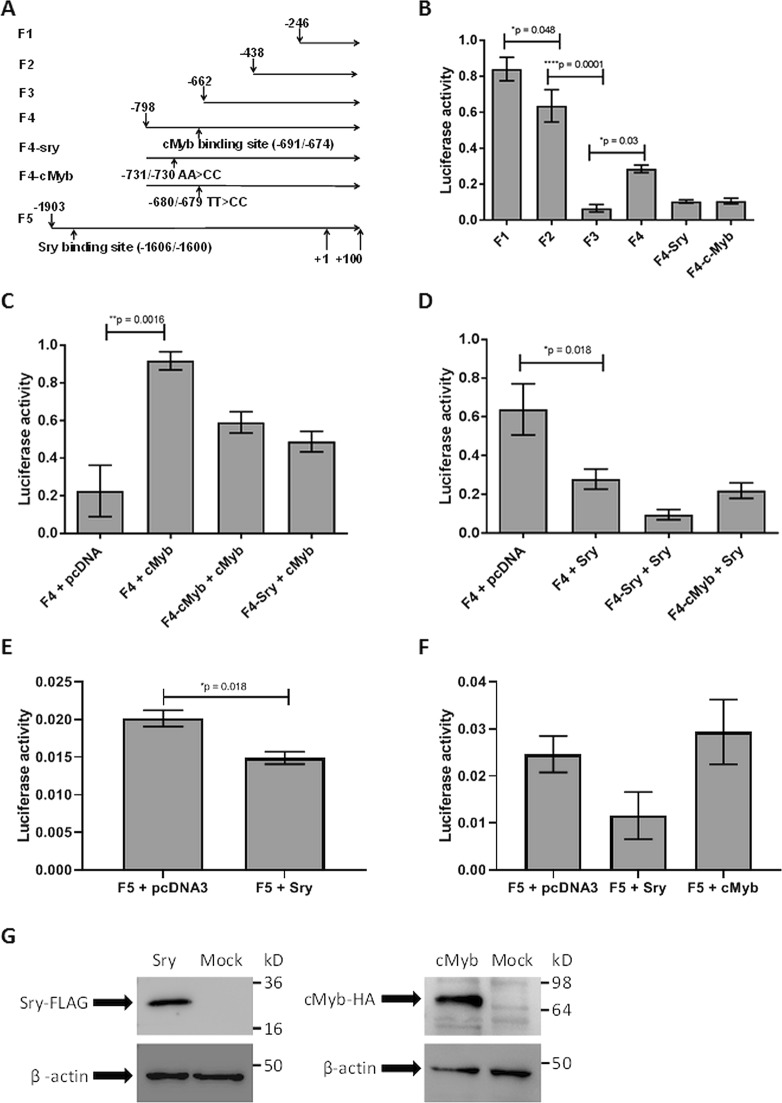
c-Myb increases and SRY decreases the luciferase activity of the *RANKL* promoter region. **a** Different lengths of the *RANKL* proximal promoter region (F1, F2, F3, F4, F5) were cloned into the luciferase reporter vector pGL3-basic. Mutations were induced in the predicted SRY binding site (−731 AA > CC) and the predicted c-Myb binding site (−680 TT > GG) in the F4 vector using site-directed mutagenesis. **b** HOS (human bone osteosarcoma) cells were transfected with pGL3-F1/-F2/-F3/-F4/-F4-SRY/-F4-c-Myb and pRL normalization vector. Luciferase activities were measured 24 h after transfection. **c** Luciferase assays of HOS cells cotransfected with the pGl3-F4 *RANKL* promoter region and pcDNA3 (empty) or c-Myb-HA and cotransfected with the mutated pGl3-F4 *RANKL* promoter regions and c-Myb-HA. **d** Luciferase assays of HOS cells cotransfected with the pGl3-F4 *RANKL* promoter region and pcDNA3 (empty) or Sry-FLAG and cotransfected with the mutated pGl3-F4 *RANKL* promoter regions and Sry-FLAG. **e** Luciferase assays of HOS cells cotransfected with the pGl3-F5 *RANKL* promoter region and pcDNA3 (empty) or Sry-FLAG. **f** Luciferase assays of human primary osteoblasts nucleofected with pGL3-F5 *RANKL* promoter region and pcDNA3 (empty) or Sry-FLAG or c-Myb-HA. **g** Western blotting of human bone osteosarcoma cells after transfection with Sry-FLAG or c-Myb-HA expression vector and of empty human bone osteosarcoma cells. All of the data are presented as the mean luciferase activities after normalization ± SEM. Asterisks indicate significant differences between luciferase activities

In this study, we aimed to characterize the proximal promoter region of the *RANKL* gene. First, to analyze the contributions of particular regions of the 5’-proximal promoter of the *RANKL* gene to its activation, various lengths of the proximal promoter region were cloned in front of the luciferase reporter gene (pGl3-Basic), and luciferase activity was measured in the HOS cells (Fig. 1a). These data showed that the F2 region (−438 bp to +100 bp) has 20% lower luciferase activity (*p* = 0.048) compared to that of the F1 region (−246 bp to +100 bp), and the F3 region (−662 bp to +100 bp) has 57% lower luciferase activity (*p* = 0.0001) compared to that of the F2 region. This result suggests that the region from −662 to −246 contains binding sites for transcriptional repressors. Surprisingly, the luciferase activity of the F4 region (−798 bp to +100 bp) was increased by 22% compared to that in the F3 region (*p* = 0.03), which suggested that the region from −798 to −662 of the *RANKL* gene contains binding sites for transcriptional activators.

Next, to identify potential transcription factors that bind to the *RANKL* proximal promoter, the F4 region of the *RANKL* promoter (Fig. 1a) was analyzed using the TF SEARCH program. SRY (possibly binding to position −732/−726 bp upstream of the *RANKL* transcription start; sequence AAACTAA; score 94.5) and c-Myb (possibly binding to position −691/−674 bp upstream of the *RANKL* transcription start; sequence TTTCCTGACTGTTGGGTG; score 85.2) were identified as probable candidates. To examine whether SRY or c-Myb putative binding sites contribute to *RANKL* promoter activity, the putative binding sites in the F4 region were mutated. At the putative SRY binding site, mutation −731/−730 AA > CC was created, and at the putative c-Myb binding site, mutation −680/−679 TT > CC was created (35–37). The results of the luciferase assays showed that the mutations introduced at the putative c-Myb (−691/−674) or SRY (−732/−726) binding sites abolished the effects of region −798/−622, as the promoter activity decreased to the activity of the F3 region (Fig. 1b), indicating that both SRY and c-Myb binding sites are important regulatory regions in the *RANKL* proximal promoter.

### SRY represses the activity of the *RANKL* proximal promoter

Next, we examined whether SRY affects the activity of the *RANKL* proximal promoter by measuring the luciferase activity of the F4 region of the *RANKL* promoter in the presence of the overexpressed SRY protein (pcDNA3-hSry) in HOS cells. In the presence of SRY, the activity of the F4 region of the *RANKL* proximal promoter decreased compared to that of the empty vector. However, mutation of the putative SRY binding site (−731/−730) did not alleviate the inhibitory action of SRY (Fig. 1d). In contrast, the luciferase activity of the mutated promoter region was lower than in the presence of the F4 *RANKL* promoter region, which suggested that another site is important for SRY regulation of *RANKL* promoter activity. We identified another possible SRY binding site, 5-[AT]AACAA[AT]-3, at position −1606/−1600 in the proximal *RANKL* promoter region.

**Fig. 2:**
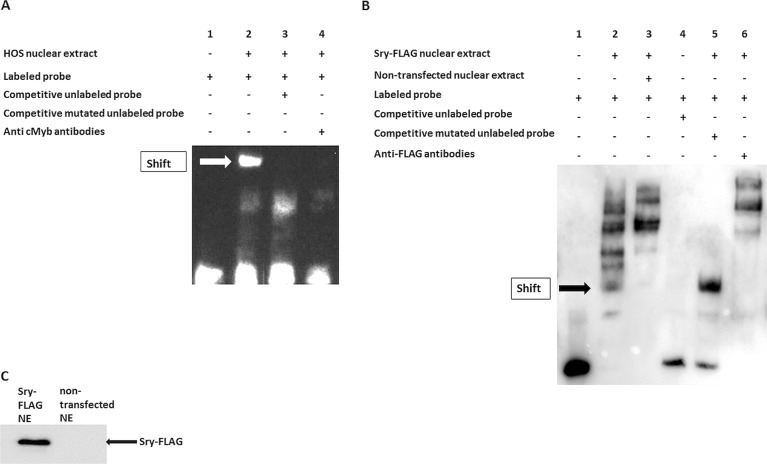
c-Myb binds to the site −691/−674 bp upstream of the *RANKL* transcription start, and SRY binds to the site −1606/−1600 bp upstream of the *RANKL* transcription start. **a** Electrophoretic mobility shift assays using human bone osteosarcoma cell nuclear extracts and biotinylated oligonucleotide probes containing site −691/−674 bp upstream of the *RANKL* transcription start. Lane 1, negative control without nuclear extract; lane 2, nuclear extract added; lane 3, competitive unlabeled probes added; lane 4, anti-c-Myb antibodies added; lane 5, mutated competitive unlabeled probes added. **b** Electrophoretic mobility shift assays using human bone osteosarcoma cell nuclear extracts and biotinylated oligonucleotide probes containing site −1606/−1600 bp upstream of the *RANKL* transcription start. Lane 1, negative control without nuclear extract; lane 2, Sry-FLAG transfected nuclear extract added; lane 3, nontransfected nuclear extract added; lane 4, competitive unlabeled probes added; lane 5, mutated competitive unlabeled probes added; lane 6, anti-FLAG antibodies added. **c** Western blotting confirmation of Sry-FLAG transfection in the nuclear extract (NE) used for the electrophoretic mobility shift assays in **b**

**Fig. 3:**
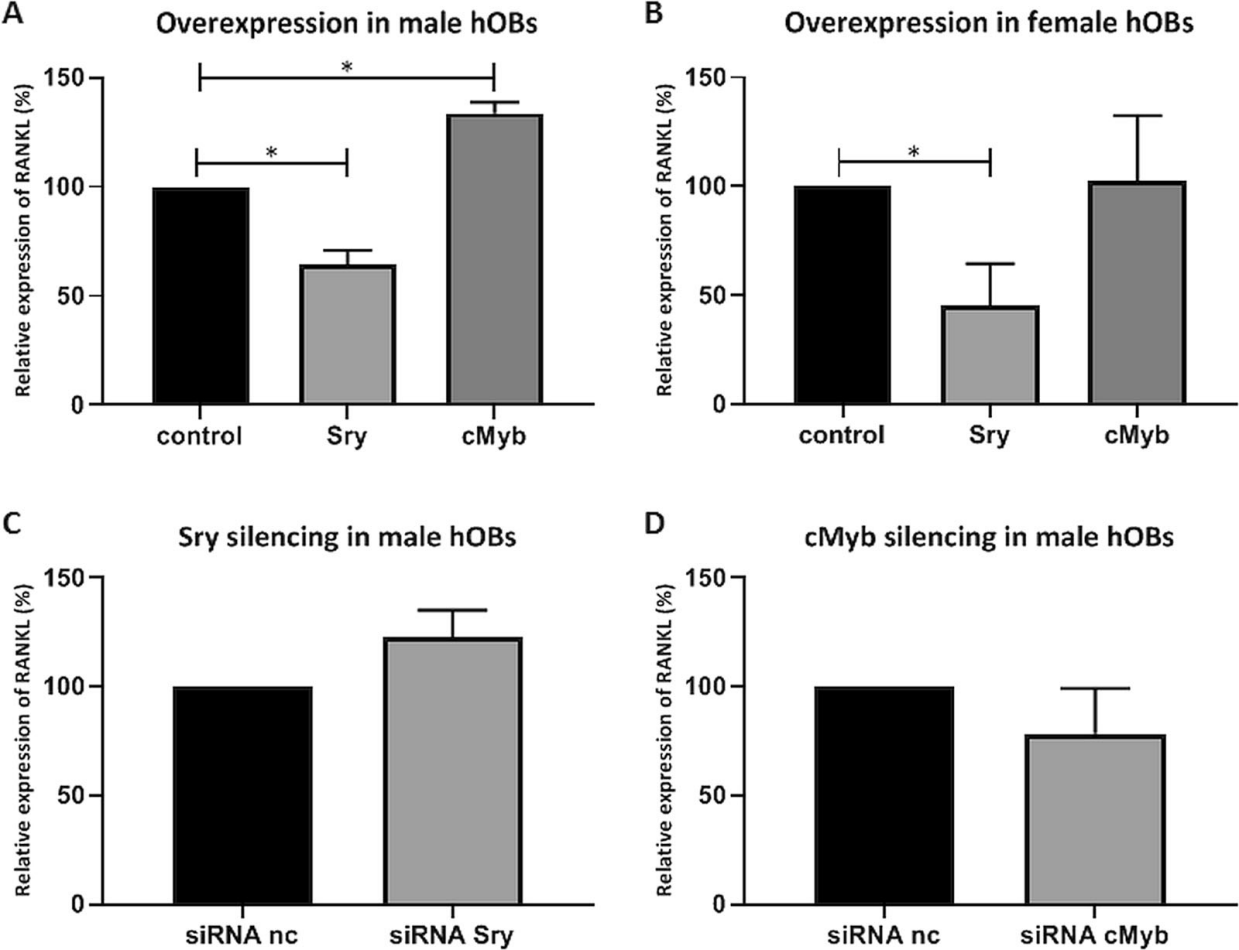
c-Myb and SRY affect the expression of the *RANKL* gene in human primary osteoblasts. **a**, **b** Female (**a**) and male (**b**) human primary osteoblasts were transfected with empty expression vector (ctl) and Sry-FLAG or c-Myb-HA. *RANKL* gene expression was measured at 48 h after treatment using q-PCR. **c**, **d** Male human primary osteoblasts were nucleofected with negative control siRNA and siRNAs against c-Myb (**c**) or Sry (**d**). *RANKL* gene expression was measured at 72 h after treatment using q-PCR. All of the data are presented as the mean relative *RANKL* expression after normalization ± SEM. Asterisks indicate significant differences between two samples. Data are representative of at least three independent experiments

Next, we examined whether SRY affects the activity of a longer *RANKL* proximal promoter region by measuring the luciferase activity of the longer proximal *RANKL* promoter region (F5 region, from −1669 to +100) in the presence of the overexpressed SRY protein (pcDNA3-hSry) in HOS cells. In the presence of SRY, the activity of the F5 region of the *RANKL* proximal promoter decreased by 30%, which suggested that this site is important for *RANKL* promoter regulation (Fig. 1e). To verify that SRY affects the *RANKL* promoter in human primary cells, human POB were also employed in the luciferase assay, resulting in the same effect (Fig. 1f), which suggests that SRY decreases *RANKL* promoter activity, regardless of the host cells, implying the role of SRY in the regulation of *RANKL* expression. Indeed, the importance of the −1606/−1600 SRY binding site was further confirmed by EMSA (Fig. 2) and silencing experiments (Fig. 3). Western blotting was used to confirm the presence of overexpressed SRY in HOS cells (Fig. 1g).

### c-Myb increases the activity of the *RANKL* proximal promoter

After identification of the putative c-Myb binding site in the RANKL proximal promoter region, we investigated whether c-Myb impacts the activity of the *RANKL* promoter. We cotransfected HOS cells with the c-Myb expressing vector (pcDNA3-c-Myb) and the pGl3-F4 reporter vector. In the presence of c-Myb, the activity of the F4 *RANKL* promoter increased 4-fold compared to that of the empty vector. Mutation in the putative c-Myb binding site (−680/−679 TT > CC) partially decreased the *RANKL* promoter activity, which indicated that c-Myb activates the RANKL promoter (Fig. 1c, f). Western blotting was used to confirm the presence of overexpressed c-Myb in HOS cells (Fig. 1g).

### c-Myb and SRY bind to the *RANKL* proximal promoter region

Electrophoretic mobility shift assays were used to examine whether the transcription factor c-Myb binds to the site −691/−674 bp upstream of the *RANKL* transcription start. Figure 2a shows the appearance of the shifted biotinylated oligonucleotides in the presence of nuclear extracts from HOS cells compared to the control without nuclear extract (Fig. 2a, lanes 2, 1), which indicated that a protein from the nuclear extract binds to the oligonucleotides. The specificity of the binding was confirmed using competitive oligonucleotides without biotin in 200-fold molar excess (Fig. 2a, lane 3). The identification of c-Myb as the binding protein was confirmed using an antibody against c-Myb that prevented the interaction between protein and DNA (Fig. 2a, lane 4). The effects of binding site mutation were tested with the mutated competitive oligonucleotides in 200-fold excess. The shifted biotinylated oligonucleotides indicated that the mutated unlabeled competitive oligonucleotides did not bind the c-Myb protein, which confirmed that the site −691/−674 bp upstream of the *RANKL* transcription start is responsible for c-Myb binding (Fig. 2a, lane 5).

Electrophoretic mobility shift assays were also used to determine whether the transcription factor SRY binds to the site −1606/−1600 bp upstream of the *RANKL* transcription start site. Figure 2b shows the appearance of the shifted biotinylated oligonucleotides in the presence of the nuclear extracts from Sry-FLAG-transfected HOS cells compared to the control without the nuclear extract (Fig. 2b, lanes 2, 1), which indicated that a protein from the transfected nuclear extract binds to the oligonucleotides. The specificity of the binding was confirmed using nontransfected nuclear extracts (Fig. 2b, lane 3) and using competitive oligonucleotides without biotin in 200-fold molar excess (Fig. 2b, lane 4). The effects of binding site mutations were tested with mutated competitive oligonucleotides in 200-fold excess. The shifted biotinylated oligonucleotides indicated that the mutated unlabeled competitive oligonucleotides did not bind the Sry-FLAG protein, which confirmed that the site −1606/−1600 bp upstream of the *RANKL* transcription start is responsible for the SRY binding (Fig. 2b, lane 5). The identification of Sry-FLAG as the binding protein was confirmed using an antibody against FLAG, which caused a supershift (Fig. 2b, lane 6). Western blotting was used to confirm the presence of Sry-FLAG in the nuclear extract used for EMSA (Fig. 2c).

Our results demonstrate that both c-Myb and SRY directly bind to the *RANKL* proximal promoter region and could consequently directly impact *RANKL* gene expression.

### Overexpression of *Sry* in human female osteoblasts lowers the expression of *RANKL*

As c-Myb increased and SRY decreased the activity of the *RANKL* promoter in our luciferase assays (Fig. 1) and as both of these transcription factors bind to the *RANKL* proximal promoter (EMSA; Fig. 2), we hypothesized that these transcription factors affect the expression of the *RANKL* gene in primary human osteoblasts. To test this hypothesis, Sry-FLAG and c-Myb-HA were transfected into male and female human primary osteoblasts, and at 48 h after transfection, *RANKL* expression was measured. As shown in Fig. 3a, overexpression of Sry decreased and overexpression of c-Myb increased expression of the *RANKL* gene in male human osteoblasts. In female human osteoblasts (Fig. 3b), overexpression of Sry decreased expression of the *RANKL* gene. These results indicated that SRY and c-Myb can regulate *RANKL* expression in human primary osteoblasts. To further confirm this hypothesis, RNA interference assays were performed using siRNAs against c-Myb or SRY in human primary osteoblasts. Figure 3c, d show the nonsignificant differences in expression of the *RANKL* gene upon silencing of c-Myb (Fig. 3c) and Sry (Fig. 3d). However, there was a trend of decreased expression of the *RANKL* gene upon silencing of c-Myb and increased expression of the *RANKL* gene upon silencing of Sry, which might suggest that SRY downregulates and c-Myb upregulates the expression of *RANKL* in human primary osteoblasts.

### SRY and c-Myb localize in human primary osteoblasts

**Fig. 4:**
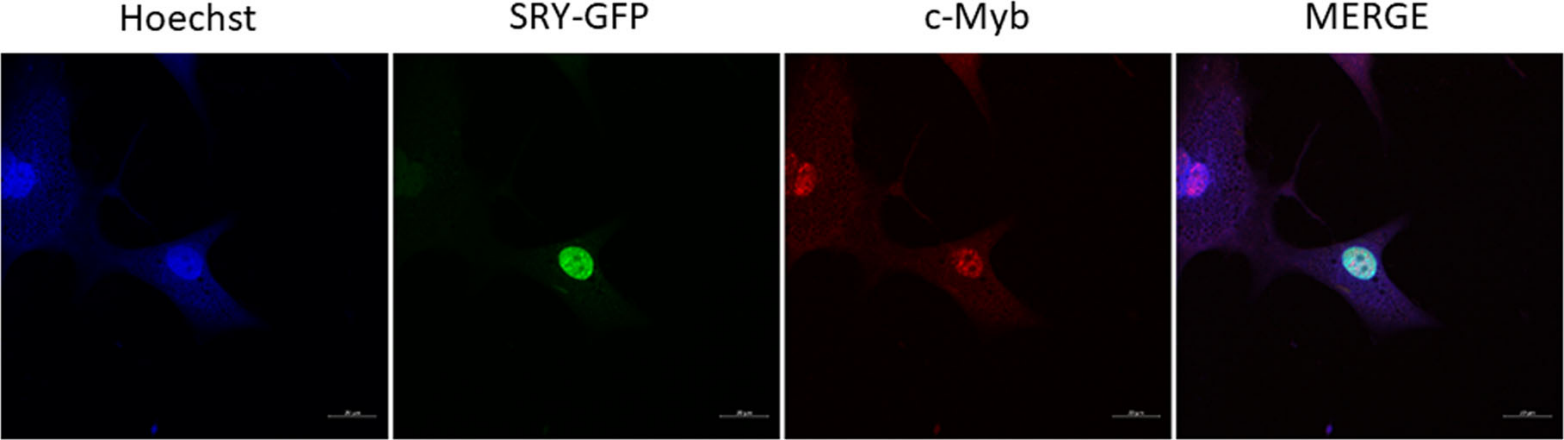
Localization of SRY and cMyb in primary osteoblast cells of male origin. Localization of SRY-GFP fusion protein in male primary osteoblasts. Color legend for merged image: blue, Hoechst 33342 nuclear stain; green, SRY-GFP fusion protein; red, antibody targeting endogenous cMyb. POB primary osteoblasts; GFP green fluorescent protein. Scale bar: 10 μm

To examine whether both transcription factors localize to the nucleus of human primary osteoblasts, which is a prerequisite for their activity on the *RANKL* promoter, we examined the subcellular localization of SRY and cMyb in primary osteoblasts of male origin. We designed a fusion between SRY and green fluorescent protein (GFP) and overexpressed the fusion protein in male primary osteoblasts. The SRY-GFP localized to the nucleus and did not show any other localization phenotype (Fig. 4, Supplementary Fig. 3). The localization of endogenous cMyb was also examined by staining with an antibody. Endogenous cMyb localized to the nucleus; however, the intensity was low. c-Myb is an oncogene, and therefore high levels would not be expected in primary cells. Our data suggest that both the transcription factors SRY and c-Myb localize to the nucleus in human primary osteoblasts, which are prerequisites the activation/inhibition of the *RANKL* promoter.

### SRY and c-Myb are present in diverse human bone cells

**Fig. 5:**
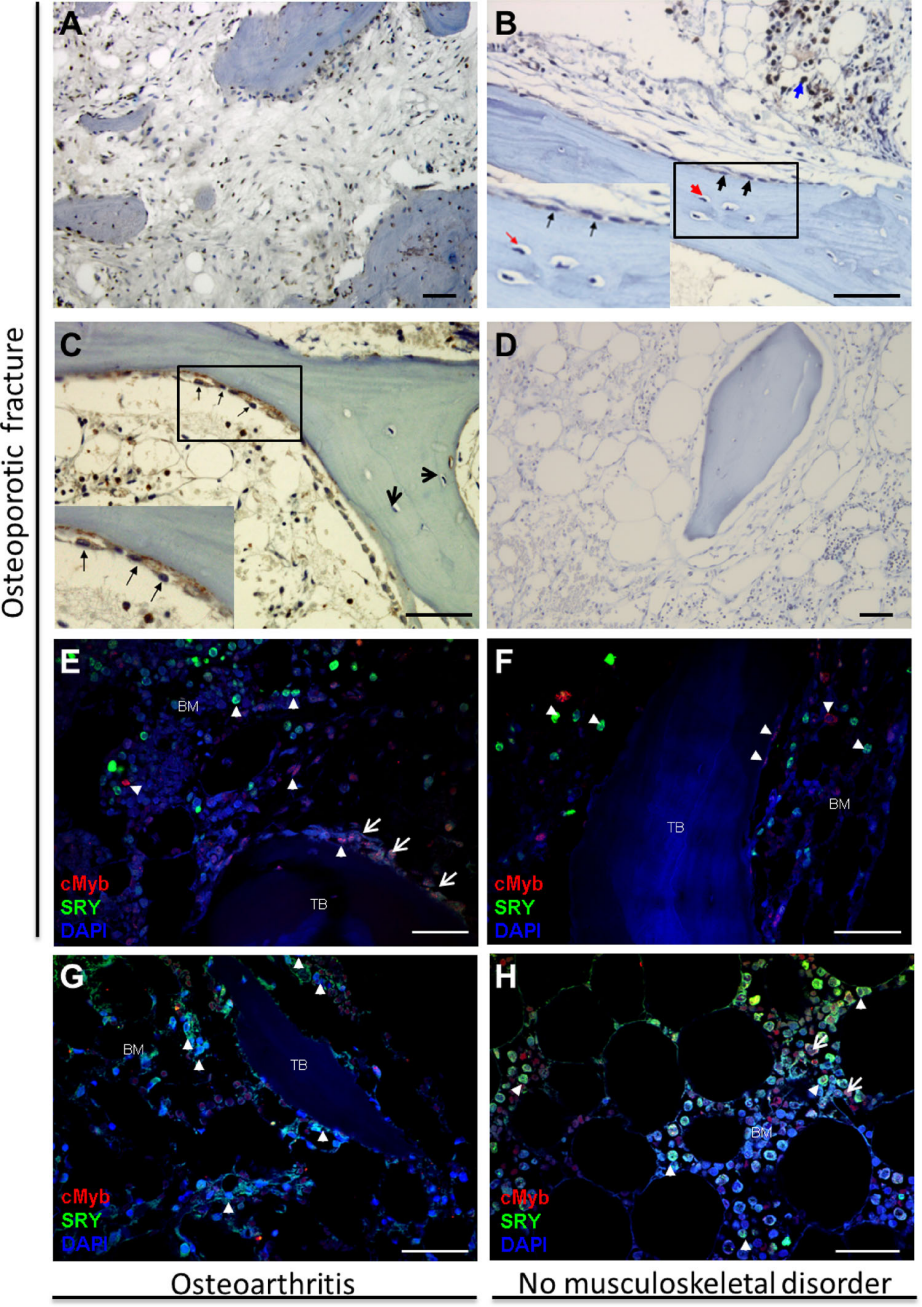
Immunolocalization of cMyb and SRY in human bone tissue. **a**–**c** Bone tissue of a male patient with osteoporotic fracture. **a** cMyb staining is observed in the nuclei of lining cells, individual bone marrow cells, and osteocytes. **b** In inactive bone, SRY staining is observed in various cells of the bone marrow (blue arrow) and in the lining cells (black arrows), while osteocytes are negative for SRY (red arrow), similar to what was observed with cMyb. **c** In bone with active processes of bone remodeling, in addition to the cells in bone marrow, SRY was observed in active osteoblasts (closed arrows) and osteocytes (open arrows) just beneath the trabecular bone surface, which suggests its role in bone remodeling via RANKL. **d** No positive SRY staining was observed in female patients with osteoporotic fracture. **e**, **f** Bone tissue of a male patient with osteoporotic fractures showing costaining of cMyb and SRY in osteoblast-like cells (arrows) on the surface of trabecular bone (**e**); single-positive SRY or cMyb cells (arrowheads, **e**, **f**) are present in bone marrow (BM); and cMyb single-positive cells are also present on the surface of trabecular bone (TB) (**e**, **f**). **g**, **h** Costaining for SRY and cMyb in bone tissue of a male patient with osteoarthritis (**g**) and in a male donor with no musculoskeletal disease (**h**) show similar patterns of localization; i.e., SRY single-positive cells (arrowheads) and SRY and cMyb double-positive cells (arrows) in bone marrow (BM). Immunohistochemistry (**a**–**d**) and double immunofluorescence (**e**–**h**) of human bone tissue. *N* = 3 donors per group (fracture, osteoarthritis and control group). Scale bars: 50 µm

Since SRY and c-Myb impacted *RANKL* promoter activity and gene expression in human primary osteoblasts and HOS cells, we investigated whether these proteins are present in physiologically relevant human bone tissues where they could actually influence the bone phenotype. To this end, we studied the localization of c-Myb and SRY in human bone tissues after an osteoporotic fracture using immunohistochemistry (Fig. 5). Intensive c-Myb staining was observed in the nuclei of lining cells and in individual bone marrow cells, but very little staining was observed in osteocytes (Fig. 5a). In dormant bone, intensive SRY staining was observed in various cells of the bone marrow (Fig. 5b, blue arrow) and with less staining of the lining cells (Fig. 5b, black arrows), while osteocytes were mostly negative for SRY (Fig. 5b, red arrow), similar to the results observed for c-Myb. During the process of active bone remodeling, SRY was observed in bone marrow cells, in active osteoblasts (Fig. 5c, black arrows), and in the osteocytes located just beneath the trabecular bone surface (Fig. 5c, red arrows), which suggested a role for SRY in bone remodeling involving RANKL. No positive SRY staining was observed in female patients (Fig. 5d), which confirmed the specificity of the SRY antibody. Double staining of SRY and c-Myb using human tissue samples from male patients with osteoporotic fractures (Fig. 5e, f) and osteoarthritis (Fig. 5g) or no such disorder (i.e., controls, Fig. 5h) showed double-positive and single-positive cells. The SRY and c-Myb double-positive cells were observed in osteoblast-like cells (Fig. 5e, arrows) on the surface of trabecular bone, while single-positive SRY or c-Myb cells (Fig. 5e, f, arrowheads) were present in bone marrow, and c-Myb single-positive cells were also present on the surface of trabecular bone (Fig. 5e).

**Fig. 6:**
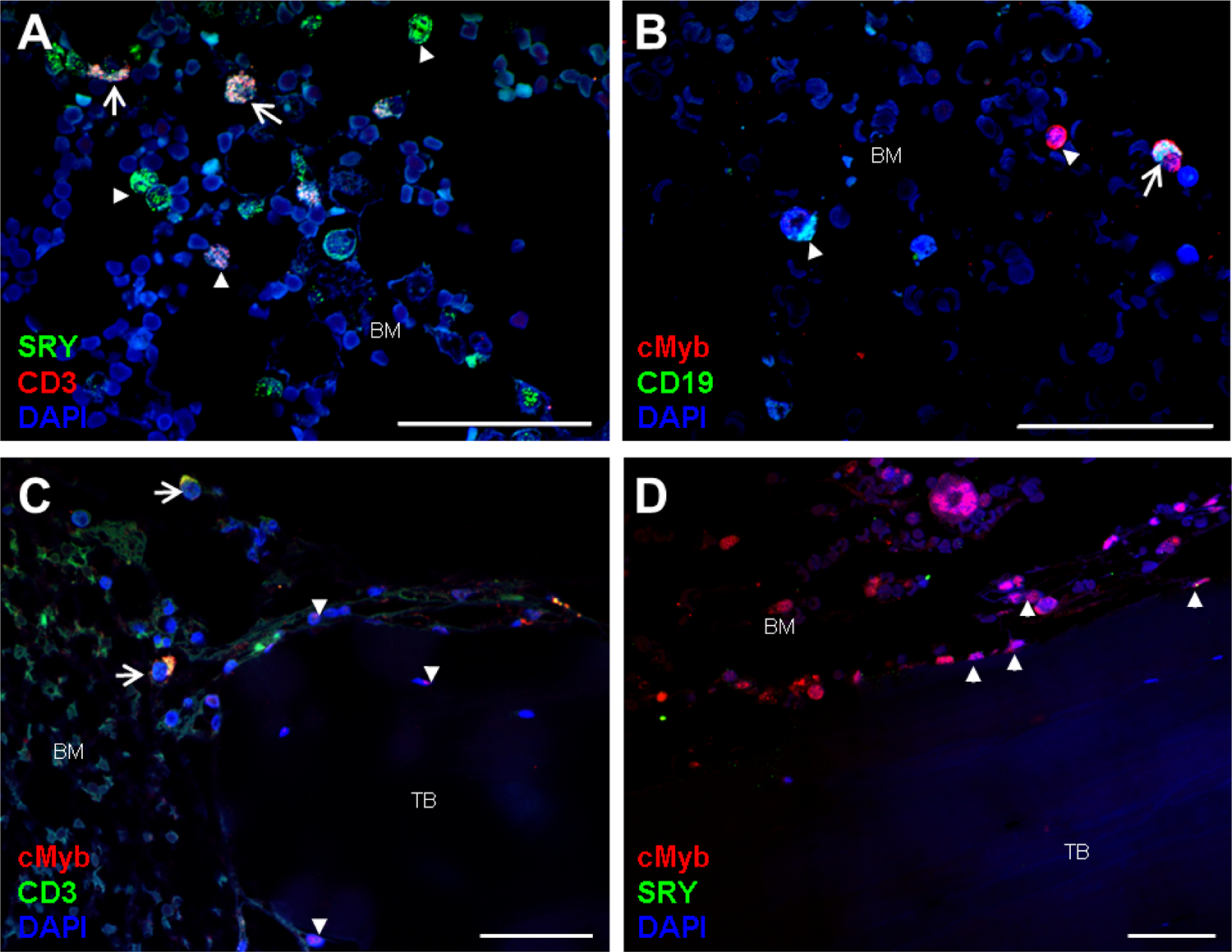
Colocalization of SRY and cMyb with T-lymphocyte and B-lymphocyte markers in human bone tissue. **a** Bone tissue of a male patient with osteoporotic fracture showing costaining of SRY with the T-lymphocyte marker CD3 (arrow) in bone marrow (BM) cells. Double-positive cells (arrows) and single-positive cells for SRY or CD3 (arrowhead) are observed within bone marrow cells. **b** Bone tissue of a male patient with osteoporotic fracture showing costaining of cMyb with the B-lymphocyte marker CD19. Double-positive cells (arrow) and single-positive cells for cMyb or CD19 (arrowhead) are observed within bone marrow (BM) cells. **c** Bone tissue of a male patient with osteoporotic fracture showing costaining of cMyb with the T-lymphocyte marker CD3 (arrow) in bone marrow (BM) cells. Double-positive cells (arrows) are observed within bone marrow cells. Single-positive cells for cMyb (arrowhead) are observed within bone marrow cells, on the trabecular bone (TB) surface, and in a few osteocytes. **d** Bone tissue of female patients with osteoporotic fracture showing negative staining for SRY and similar patterns of cMyb staining as in the male osteoporotic fracture tissue. Scale bars: 50 µm

**Fig. 7:**
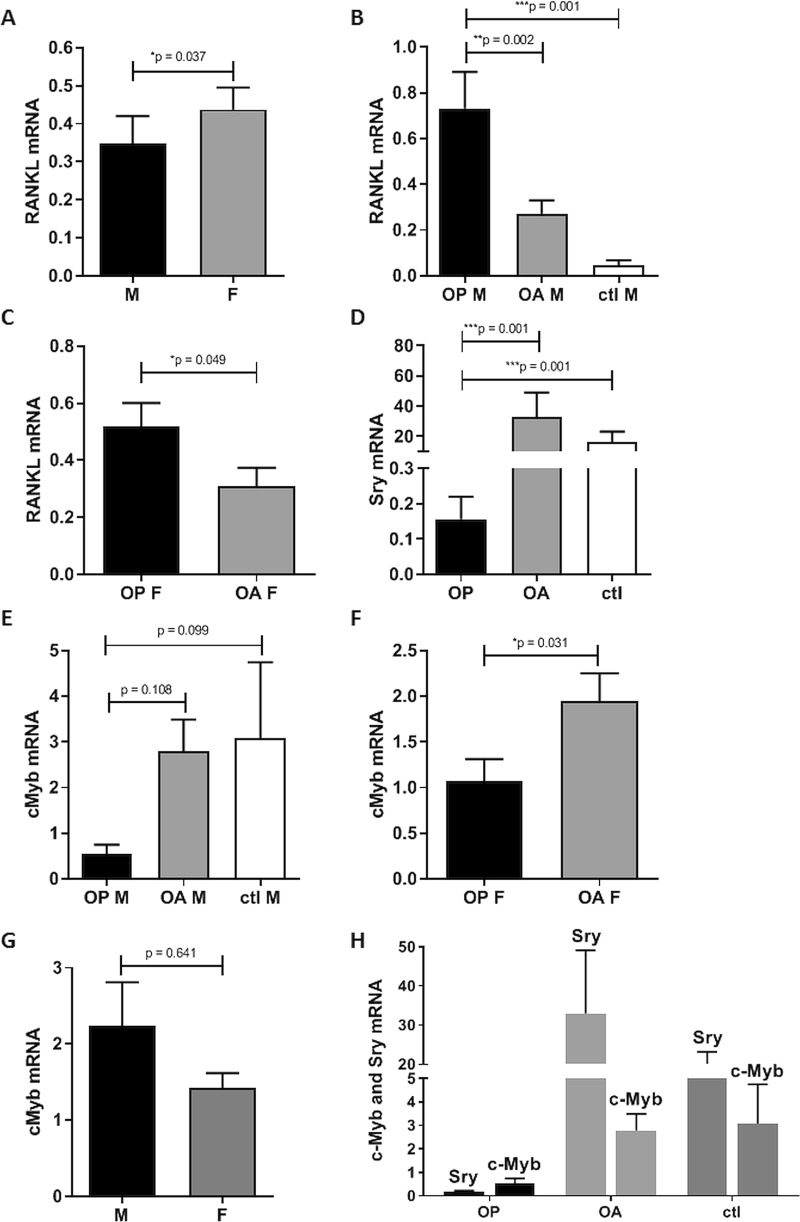
Expression of *RANKL*, c-Myb, and Sry in human bone tissues is dependent on gender and health state. qPCR was used to measure gene expression in human bone samples from male osteoporotic (OP, *n* = 12), osteoarthritic (OA, *n* = 13) and healthy (ctl. *n* = 12) patients and from female osteoporotic (OP, *n* = 46) and osteoarthritic (OA, *n* = 29) patients. Mean mRNA levels were normalized to RPLP0. Asterisk, significant differences between groups. **a** Comparison of *RANKL* gene expression in bone samples from male and female patients. **b** Comparison of *RANKL* gene expression in bone samples from OP, OA, and ctl male patients. **c** Comparison of *RANKL* gene expression in bone samples from OP and OA female patients. **d** Comparison of *Sry* gene expression in bone samples from OP, OA, and ctl male patients. **e** Comparison of c-*Myb* gene expression in bone samples from OP, OA, and ctl male patients. **f** Comparison of c-*Myb* gene expression in bone samples from OP and OA female patients. **g** Comparison of c-*Myb* gene expression in bone samples from male and female patients. **h** Comparison of *Sry*: c-*Myb* expression ratios in bone samples from OP, OA, and ctl male patients

Immunolocalization studies showed both SRY and c-Myb single-positive cells and the colocalization of these proteins with the T-lymphocyte marker CD3 in bone marrow cells (Fig. 6a, c, Supplementary Fig. 4A–D). Moreover, c-Myb colocalization with the B-lymphocyte marker CD19 was shown (Fig. 6b, Supplementary Fig. 4E–H). The results of SRY and CD19 colocalization, which required different CD19 antibodies, showed nonspecific staining of CD19 (data not shown). Bone tissue from a female patient with osteoporotic fracture showed negative staining for SRY and similar patterns of c-Myb staining as for the male osteoporotic fracture tissue (Fig. 6d). The gene expression of these markers in human bone tissue is shown in Fig. 7.

### Osteoporotic males show significantly decreased expression of *Sry* and highly increased expression of *RANKL*

As male-specific transcription factor SRY decreased the activity of the *RANKL* promoter in vitro, we hypothesized that SRY downregulates *RANKL* expression in vivo and that lower *RANKL* expression will be observed in males. To quantify the expression of *RANKL*, *Sry*, and *c-Myb* in human bone samples, real-time PCR was used (Fig. 7). Indeed, the expression of *RANKL* was 20% lower in males than in females, which supported our hypothesis (*p* = 0.037; Fig. 7a). When comparing *RANKL* expression between osteoporotic, osteoarthritic, and healthy individuals, *RANKL* was significantly increased in both male and female osteoporotic patients. Indeed, in male osteoporotic patients, *RANKL* expression was 17-fold higher than that in healthy controls (*p* = 0.001; Fig. 7b) and 3-fold higher than that in osteoarthritic patients (*p* = 0.002; Fig. 7b). In female osteoporotic patients, *RANKL* expression was almost twice that in osteoarthritic patients (*p* = 0.049; Fig. 7c). Interestingly, the expression of *RANKL* in male osteoporotic patients was 40% higher than that in female osteoporotic patients. These data indicate that the expression of *RANKL* is upregulated in male osteoporotic patients and that the expression of *RANKL* increases more in male than female osteoporotic patients, which associates the expression of *RANKL* with osteoporosis.

As our in vitro data suggested that male-specific transcription factor SRY represses the activity of the *RANKL* promoter, the expression of *Sry* was examined in osteoporotic patients and compared with healthy and osteoarthritic individuals. *Sry* expression was 210-fold lower in osteoporotic men compared to osteoarthritic men (*p* = 0.001) and 105-fold lower compared to healthy men (*p* = 0.001; Fig. 7d). These data demonstrate that *Sry* expression is significantly downregulated in osteoporotic patients. Together with the in vitro data, our results suggest that downregulation of *Sry* causes upregulation of *RANKL* and may consequently cause the development of osteoporosis in male patients. Moreover, our data suggest that SRY is one of the regulators of *RANKL* expression in males and might protect men from osteoporosis.

Given that our in vitro data indicate that *RANKL* promoter activity is controlled by the transcription factor c-Myb, we next examined the expression of *c-Myb* in osteoporotic and osteoarthritic patients and healthy individuals. The expression of *c-Myb* appeared to be lower in male osteoporotic patients compared to that in male osteoarthritic patients and healthy males; however, these data were not significant (Fig. 7e). The expression of c-Myb was half in osteoporotic than osteoarthritic females (*p* = 0.031; Fig. 7f). However, no significant differences in *c-Myb* expression were observed between male and female bone samples (*p* = 0.641; Fig. 7g). These data demonstrate that *c-Myb* is slightly downregulated in osteoporotic patients and that both female and male patients express similar levels of *c-Myb*, which indicates that the expression of *c-Myb* is not gender dependent.

Figure 7h shows the comparison of expression of *Sry* and *c-Myb* in male patients. Again, a drastic downregulation of *Sry* was observed in osteoporotic males compared to osteoarthritic and healthy individuals. Intriguingly, in osteoporotic males, the expression of *c-Myb* also appeared lower than that in osteoarthritic patients and healthy individuals; however, these data were not significant. In healthy and osteoarthritic male subjects, the expression of *Sry* was 5-fold and 12-fold higher, respectively, than the expression of *c-Myb*. On the other hand, in osteoporotic patients, the expression of *c-Myb* was 3-fold higher than the expression of *Sry*. Altogether, these data reveal that in osteoporotic male patients, the expression of *Sry* was downregulated.

## Discussion

RANKL is a protein that is involved in bone metabolism and modulates the activation and differentiation of osteoclasts. Its activation leads to increased bone resorption. In bone diseases, such as osteoporosis, *RANKL* expression is highly increased; therefore, RANKL represents a great target for osteoporosis treatment. Indeed, a monoclonal antibody against RANKL (denosumab) is currently used for osteoporosis treatment and prevention of skeletal-related events in patients with bone metastases. Playing a central role in bone remodeling, RANKL represents a good drug target for the development of novel cheaper approaches for the prevention of fragility fractures and osteoporosis. Thus, deciphering the regulation of *RANKL* gene expression is necessary. The expression of *RANKL* is regulated by transcription factors that bind to its distal and proximal promoter regions. Here, we aimed to identify transcription factors that bind to the *RANKL* proximal promoter region and can regulate its expression. We confirmed that the −662/−798 bp region in the *RANKL* proximal promoter contains binding sites for transcription factors, c-Myb and SRY. c-Myb is one of the oncogenes previously shown to be involved in bone formation and chondrogenesis^41–43^. SRY is a sex-specific transcription factor, expressed only in men and responsible for sex determination. We demonstrated that c-Myb increased and SRY decreased the activity of the *RANKL* promoter (Fig. 1). Using EMSA, we confirmed that c-Myb directly binds to the −691/−674 bp site (Fig. 2a) and showed that SRY binds to the −1606/−1600 bp region (Fig. 2b) of the *RANKL* promoter. When SRY was overexpressed in female human primary osteoblasts, the expression of *RANKL* was significantly reduced, which indicates that SRY decreases *RANKL* expression in human primary osteoblasts (Fig. 3a). Gene expression in the human bone samples revealed that in women bone tissues RANKL expression is 20% higher compared to that in men bone tissues, which suggested that higher expression of *RANKL* accounts for lower bone mineral density in women. This finding is in agreement with observations that men have higher bone mineral density^47–49^ and suffer from osteoporosis less frequently than women^50,51^. Importantly, in our osteoporotic patients, the expression of *RANKL* was highly upregulated and dependent on gender. In osteoporotic men, the expression of *RANKL* was significantly increased (17-fold). We demonstrated that the expression levels of *RANKL* were highly inversely correlated with the expression levels of *Sry*, which implies that the gender differences in the *RANKL* expression levels might be due to the sex-specific transcription factor SRY. Moreover, the drastic downregulation of *Sry* in male osteoporotic patients together with the upregulation of *RANKL* suggests that lower *Sry* expression might cause upregulation of *RANKL* and cause the development of osteoporosis in males. The mechanism behind the downregulation of Sry in men, leading to the upregulation of *RANKL* and osteoporosis, remains unknown. This study is the first to identify the gender-specific transcription factor SRY as a regulator of *RANKL* expression by directly binding to the *RANKL* promoter region. Our results indicate that SRY could be one of the reasons for gender differences in bone mineral density and for gender differences in the pathogenesis of osteoporosis. Previous studies have shown differences in the microarchitecture between osteoporotic and osteoarthritic bone^52^ could now be explained by SRY. Strong correlations between the reduced levels of *Sry* and incidence of osteoporosis in male osteoporotic patients suggest that SRY can serve as a biomarker for male osteoporosis. Moreover, our results provide a novel target for the development of new therapeutic approaches for the prevention of bone loss in men and women. Namely, by the stimulation of SRY-mediated downregulation of *RANKL* expression or by application of SRY analogs, the expression of *RANKL* could be lowered, and consequently, bone resorption prevented.

One of the major gender differences that contributes to bone phenotype and is dependent on sex is hormones. The role of hormones in bone remodeling has been well established^53^. Indeed, the decline of estrogens in postmenopausal women is associated with a decrease in bone mineral density, which indicates that lower estrogen levels influence bone mineral density^53^. Estrogen protection of bone mass is mediated via estrogen receptor α^53^. However, this mechanism is not limited to females. Estrogen metabolism has also been demonstrated to influence bone density in males, which indicates that estrogen is a regulator of bone remodeling both in males and females (reviewed in^54^). On the other hand, androgen hormones are required for the maintenance of trabecular bone in males, and their role has also been implicated in female bone remodeling^53^. Notably, RANKL-mediated increases in bone resorption have already been associated with sex hormones^55^. Although bone mass, size and quality differ between genders, no gender-distinctive transcriptional regulation mechanism that affects bone remodeling has been described to date. Given the important contribution of hormones to bone remodeling in our male patients, changes in estrogen or androgen levels might have contributed to the onset of osteoporosis. However, this explanation is less probable as patients with abnormal levels of hormones were excluded from the study. Here, we unveil the first gender-related transcriptional mechanism that distinctly regulates bone remodeling in males.

The role of SRY in bone biology is plausible because the *Sry* gene is part of the *SOX* gene family with an HMG box DNA binding domain^56,57^ involved in bone development and turnover^58–60^. Mutations of *Sry* cause XY gonadal dysgenesis (Swyer syndrome), in which a genetically male fetus fails to develop testes, which consequently leads to the development of female genitalia with a defective reproductive system. Hormonal replacement therapy is necessary to assure a normal menstrual cycle and secondary sex characteristics and to reduce the risks of osteopenia, which suggests the importance of SRY in normal bone development and hormonal status^61–63^. The tissue widespread expression of *Sry* in adult men has also been observed, which indicates functions of SRY other than the development of testes^64^. Importantly, *RANKL* upregulation and *c-Myb* downregulation were also observed in osteoporotic women (Fig. 7c, f), which suggested that in females, another transcription factor might have a similar role as SRY in males. Further studies are required to determine the transcription factors that regulate *RANKL* expression in women. We speculate that other members of the SOX family might be involved in c-Myb–dependent regulation of *RANKL* in women. Altogether, this is the first study that demonstrates the importance of SRY in the regulation of *RANKL* expression in males.

Here, we show that c-Myb activates the *RANKL* promoter by direct binding and causes upregulation of *RANKL* expression in primary osteoblasts. Moreover, we demonstrate that c-Myb is expressed in human bone tissues by immunohistochemistry and Q-PCR, suggesting the importance of c-Myb in bone biology. Indeed, it has already been shown that c-Myb is involved in bone biology and, more specifically, in novel bone formation^41–43^ and chondrogenesis^65^. In addition to the upregulation of *RANKL* in osteoporosis, dysregulation of *RANKL* has also been associated with tumorigenesis^66^ and metastases^67^ in several cancers. The importance of RANKL in tumorigenesis has been underpinned by clinical trials where denosumab decreased the risk of metastasis development^68–70^. c-Myb has recently been associated with the Wnt/β-catenin pathway as a mediator of metastasis in breast cancer^71^, and has been shown to promote migration and invasion of mammary tumor cells through regulation of cathepsin D and matrix metalloproteinase 9^72^. Our data demonstrate that c-Myb overexpression activates the *RANKL* promoter in vitro and might be the reason for the elevated levels of *RANKL* in bone cancer diseases.

In conclusion, we identified two novel transcription factors, SRY and c-Myb, that regulate the expression of *RANKL* in human primary osteoblasts and that might influence the expression of *RANKL* in vivo. Importantly, we unveiled a novel gender-specific transcriptional mechanism that contributes to the regulation of *RANKL* in a sex-specific manner and that might contribute to gender differences in bone phenotypes.

## Supplementary information

Supplementary Information.
