# Supplementary material for: Sex-determining region Y (SRY) attributes to gender differences in RANKL expression and incidence of osteoporosis
Source: Exp Mol Med. 2019 Aug 14;51(8):97. doi: 10.1038/s12276-019-0294-3 (PMC6802671; doi:10.1038/s12276-019-0294-3)
Supplement: Supplementary file 1 — Supplementary Information. [file 12276_2019_294_MOESM1_ESM.docx]

**
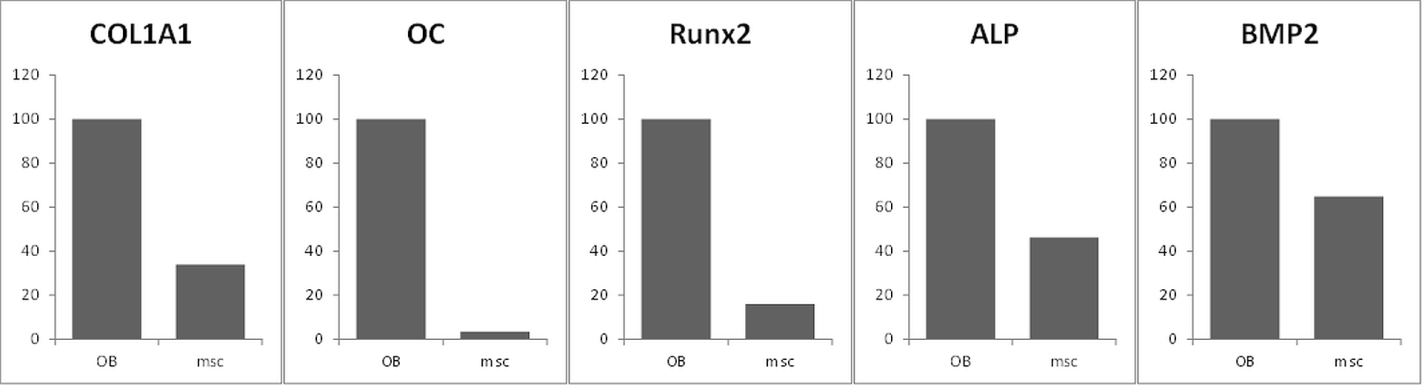
 Supplemental Figure 1: Primary human osteoblasts used in our study express markers of mature osteoblasts.**

qPCR was used to compare the expression of osteogenic genes between nondifferentiated mesenchymal stem cells (MSCs) and primary human osteoblasts (hOBs). Expression is normalized to the housekeeping gene *GAPDH*. The expression of all of the osteogenic genes is higher in hOBs than in MSCs. COL1A1, collagen type I alpha 1 chain; OC, osteocalcin; Runx2, runt-related transcription factor 2; ALP, alkaline phosphatase; BMP2, bone morphogenetic protein 2.

**
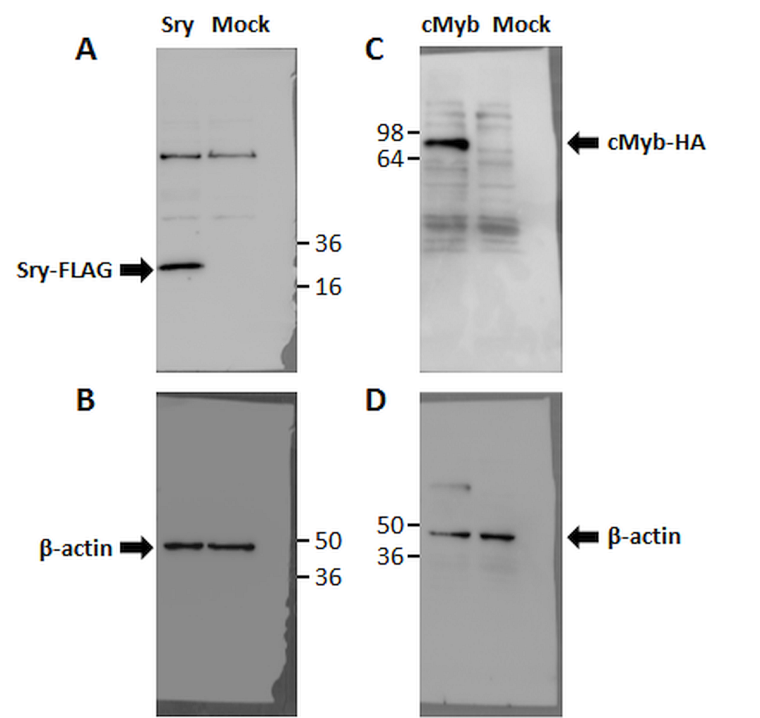
**

**Supplemental Figure 2: Full images of Western blots of HOS cells transfected with Sry-FLAG or cMyb-HA.**

Full images of Western blots used in Figure 1 Panel G. (A) HOS cells transfected with Sry-FLAG and empty HOS cells. (B) β –actin loading controls (C) HOS cells transfected with cMyb-HA and empty HOS cells. (D) β –actin loading controls loading controls for panel C

**
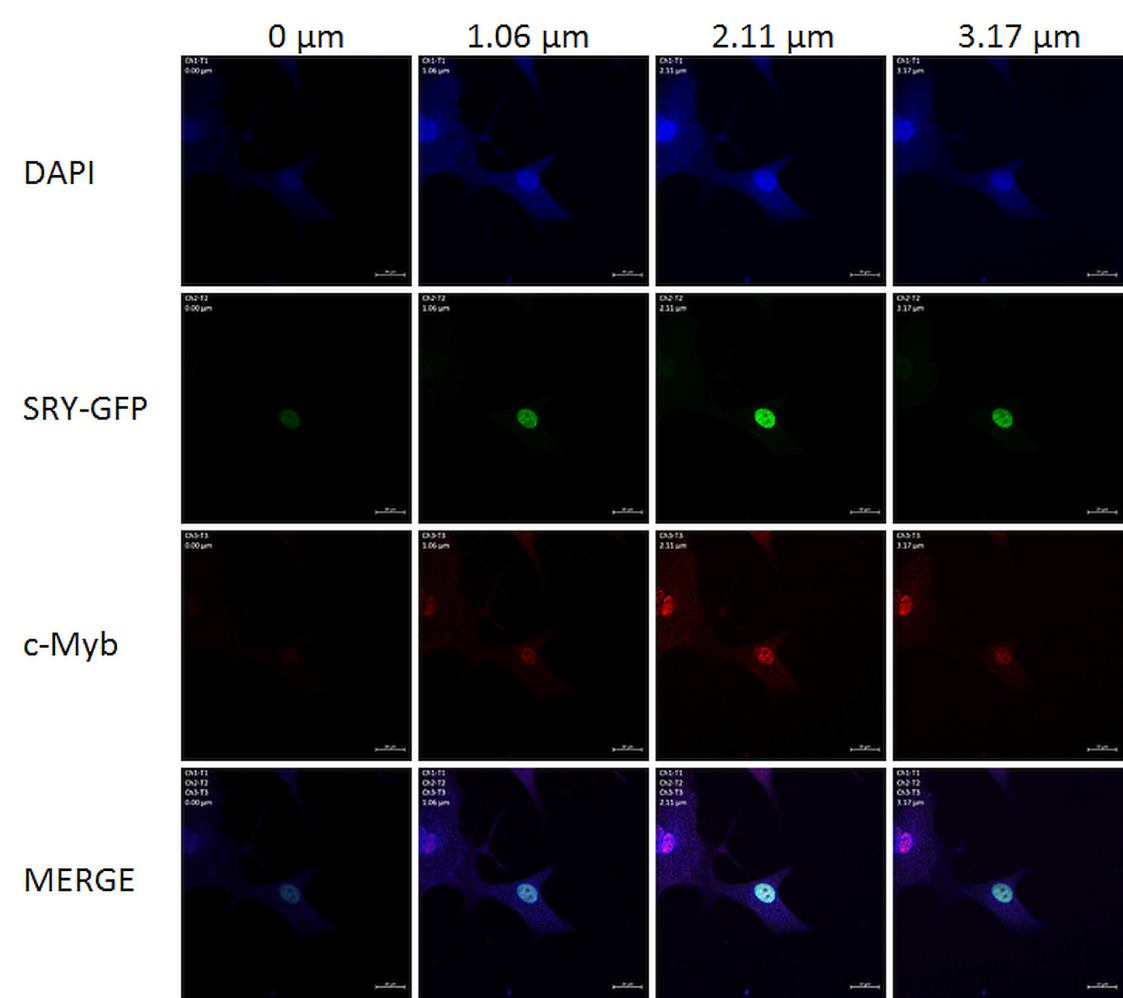
**

**Supplemental Figure 3. Z-stack confocal microscopy images of human primary osteoblasts transfected with SRY-GFP.** Color legend for merged image: blue, Hoechst 33342 nuclear stain; green, SRY-GFP fusion protein; red, antibody targeting endogenous cMyb. (POB, primary osteoblasts; GFP, green fluorescent protein. Scale bar: 10 μm.

**
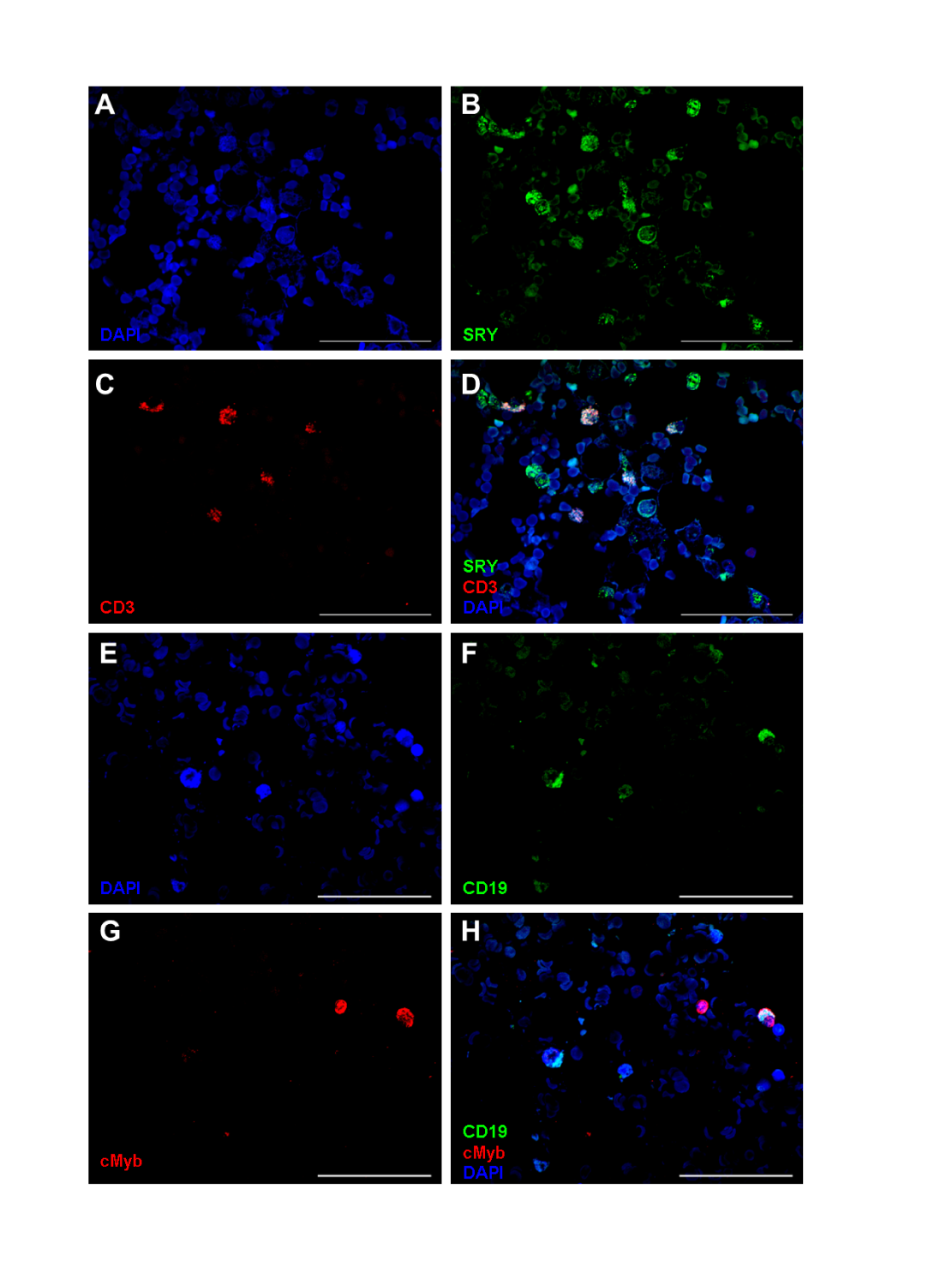
**

**Supplemental Figure 4. Colocalization of SRY and cMyb with T-lymphocyte and B-lymphocyte markers in human bone tissue.**

Bone tissue of male patients with osteoporotic fracture showing costaining of SRY with the T-lymphocyte marker CD3 in bone marrow cells (D). Single channels for DAPI (A), SRY (B) and CD3 (C) are shown. Bone tissue of a male patient with osteoporotic fracture showing costaining of cMyb with the B-lymphocyte marker CD19 (H) and respective single channels for DAPI (E), CD19 (F) and C-Myb (G). Scale bars: 50 µm.

**Supplemental Table 1. Descriptive statistics of 112 patients enrolled in the study.** All of the patient's data in Supplemental Table 1 are presented as the means and 95% confidence intervals of the means, except gender, which is presented as women (W) to men (M) ratio.
Legend: OA (osteoarthritis), OP (osteoporosis), CTL (control), BMI (body mass index in kg/m^2^), BMD (bone mineral density in g/cm^2^), NA (not available).

|  | OA | OP | CTL |
| --- | --- | --- | --- |
| Age (years) | 71,7 (69,4 to 73,9) | 75,7 (73,7 to 77,6) | 68,0 (61,4 to 74,6) |
| Gender (W to M ratio) | 46 : 12 | 29 : 13 | 0 : 12 |
| BMI | 29,3 (27,7 to 30,8) | 25,0 (24,0 to 26,0) | 25,3 (22,9 to 27,6) |
| Hip BMD | 0,899 (0,846 to 0,952) | 0,701 (0,655 to 0,748) | NA |
| Hip t-score | -0,756 (-1,141 to -0,370) | -2,263 (-2,603 to -1,923) | NA |
| Femoral neck BMD | 0,785 (0,734 to 0,836) | 0,600 (0,566 to 0,633) | NA |
| Femoral neck t-score | -1,239 (-1,672 to -0,807) | -2,706 (-3,002 to -2,410) | NA |
| Lumbar spine BMD | 1,013 (0,950 to 1,076) | 0,847 (0,790 to 0,904) | NA |
| Lumbar spine t-score | -0,469 (-1,009 to 0,070) | -1,917 (-2,424 to -1,410) | NA |

**Supplemental Table 2. Primers and oligonucleotides used in the study.**

| **Name** | **Description** | **Forward primer** | **Reverse primer** |
| --- | --- | --- | --- |
| F1 -246/+100 (346 bp) | Amplification of F1 region of the RANKL proximal promoter | CGGAGATCTCCAGAAAGACAGCTGAGGATGG | CCAAAGCTTCTTGTCTGCGGCCAACTC |
| F2 -438/+100 (538 bp) | Amplification of F2 region of the RANKL proximal promoter | CCGAGATCTAGAGGTTGGACAGGAAGGGTC |  |
| F3 -662/+100 (762 bp) | Amplification of F3 region of the RANKL proximal promoter | ACGAGATCTATGCTTGCTTCTGGCTACACGC |  |
| F4 -798/+100 (898 bp) | Amplification of F4 region of the RANKL proximal promoter | GCCAGATCTCACTAAGAGCCACAGTTCTGAATAGAGG |  |
| F5 -1669/+100 (1769 bp) | Amplification of F5 region of the RANKL proximal promoter | GCCAGATCTTGAGGAAGAAGAGGAGGAG |  |
| F4 c-Myb mutation | Used for mutation of RANKL promoter in putative c-Myb binding site | TCTTTCCTGACTG**GG**GGGTGAGCCCTCC | GGAGGGCTCACCC**CC**CAGTCAGGAAAGA |
| F4 Sry mutation | Used for mutation of RANKL promoter in putative Sry binding site | TTCTTTAGCAATGA**CC**CTAACATTTAACTG | CAGTTAAATGTTAG**GG**TCATTGCTAAAGAA |
| pcDNA3-FLAG-hSRY | Used for removal of FLAG tag from pcDNA3-FLAG-hSRY | GGATCCGCCACCCATTGACTACAAAGACGTTGACGACAAGATGCA | TGCATCTTGTCGTCAACGTCTTTGTAGTCAATGGGTGGCGGATCC |
| pEGFP-SRY | Used for amplification of the SRY open reading frame from plasmid pcDNA3-FLAG-hSRY | GGATCCGCCACCCATTGACTACAAAGACGTTGACGACAAGATGCA | TGCATCTTGTCGTCAACGTCTTTGTAGTCAATGGGTGGCGGATCC |
| c-Myb EMSA | Used for hybridization for c-Myb EMSA | [btn]CATCTCTTTCCTGACTGTTGGGTGAGCCCT | [btn]AGGGCTCACCCAACAGTCAGGAAAGAGATG |
| Sry EMSA | Used for hybridization for Sry EMSA | [btn]TTCTTTAGCAATGAAACTAACATTTAACTG | [btn]CAGTTAAATGTTAGTTTCATTGCTAAGAA |
| Sry EMSA 2 | Used for hybridization for Sry EMSA 2 | [btn]CCCAAAGTTAACAAACAAAAAGTGGGAAGA | [btn]TCTTCCCACTTTTTGTTTGTTAACTTTGGG |
| RANKL qPCR | Used for qPCR analysis of RANKL gene expression | TGATTCATGTAGGAGAATTAAACAGG | GATGTGCTGTGATCCAACGA |
| RPLP0 qPCR | Used for qPCR analysis of RPLP0 gene expression for normalization | TCTACAACCCTGAAGTGCTTGAT | CAATCTGCAGACAGACACTGG |
| GAPDH qPCR | Used for qPCR analysis of RPLP0 gene expression for normalization | TGCACCACCAACTGCTTAGC | TGGCATGGACTGTGGTCATG |
| Sry qPCR | Used for qPCR analysis of Sry gene expression | GGGATGACTGTACGAAAGCC | TCTTTGTAGCCAATGTTACCCG |
| COL1A1 qPCR | Used for qPCR analysis of COL1A1 gene expression | GCCAAGACGAAGACATCCCA | GTTTCCACACGTCTCGGTCA |
| OC qPCR | Used for qPCR analysis of OC gene expression | AGCGAGGTAGTGAAGAGAC | GAAAGCCGATGTGGTCAG |
| Runx2 qPCR | Used for qPCR analysis of Runx2 gene expression | AGCAAGGTTCAACGATCTGAGAT | TTTGTGAAGACGGTTATGGTCAA |
| ALP qPCR | Used for qPCR analysis of ALP gene expression | CCAAGTACTGGCGAGACCAA | GTGGAGACACCCATCCCATC |
| BMP2 qPCR | Used for qPCR analysis of BMP2 gene expression | GGGCATCCTCTCCACAAA | GTCATTCCACCCCACGTC |
